# Self-organizing "harmonic dominance stripes" in a spiking network model of the auditory system

**DOI:** 10.1186/1471-2202-14-S1-P52

**Published:** 2013-07-08

**Authors:** Marcos A Cantu

**Affiliations:** 1 Graduate Program for Neuroscience, Boston University, Boston, MA, 02215, USA; 2 Center for Computational Neuroscience and Neural Technology Boston University, Boston, MA, 02215, USA

## 

**Figure 1:**
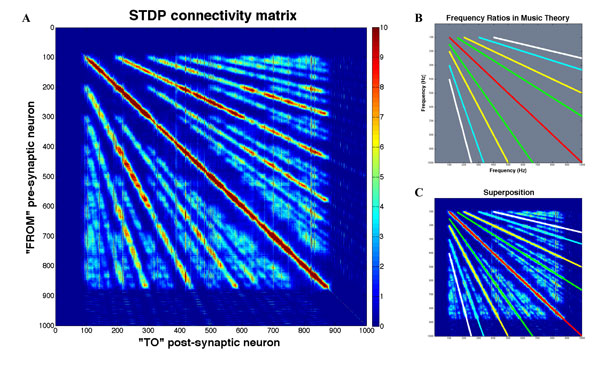
**Self-Organizing "Harmonic Dominance Stripes" in a Spiking Network Model of the Auditory System **A**: Connectivity Matrix**. After 500 trials, self-organizing "harmonic dominance stripes" had become clearly visible in the connectivity matrix between the two layers of neurons. Besides the "tonotopic" unison interval stripe along the diagonal, stripes corresponding to the octave and fifth intervals in Western music theory (see Figure 1B and 1C) were also clearly visible. The colorbar on the Right shows the strength of synaptic connections between neurons. **B**: Frequency ratios from 100 to 1000 Hz for Unison (red), Octave (yellow and white) and Perfect Fifth (green and cyan) intervals in Western music theory. **C**: Unison, Octave and Perfect Fifth Frequency ratios from Figure 1B superimposed over the STDP connectivity matrix from Figure 1A.

A model of the auditory system was developed which implements spike timing dependent plasticity (STDP) [1] in a biologically realistic network of spiking excitatory Izhikevich neurons [2]. The model self-organizes in response to periodic sound and exhibits "harmonic dominance stripes" (Figure 1A) in the STDP connectivity matrix between the two layers of the network. These "harmonic dominance stripes" are akin to the activity dependent formation of "ocular dominance columns" in the visual system, as both are architectural features that emerge through a process of self-organization as a result of passive exposure to patterned sensory input during development. The self-organizing "harmonic dominance stripes" also happen to correspond to consonant intervals (Figure. 1B and 1C) in music theory.
